# Enhancing Exposure Treatment for Youths With Chronic Pain: Co-design and Qualitative Approach

**DOI:** 10.2196/41292

**Published:** 2023-03-09

**Authors:** Lea Schemer, Courtney W Hess, Amanda R Van Orden, Kathryn A Birnie, Lauren E Harrison, Julia A Glombiewski, Laura E Simons

**Affiliations:** 1 Department of Clinical Psychology and Psychotherapy University of Kaiserslautern-Landau Landau Germany; 2 Department of Anesthesiology, Perioperative, and Pain Medicine Stanford University School of Medicine Palo Alto, CA United States; 3 Department of Anesthesiology, Perioperative, and Pain Medicine Department of Community Health Sciences Alberta Children’s Hospital Research Institute, University of Calgary Calgary, AB Canada

**Keywords:** co-design, participatory design, pain, exposure treatment, youths with chronic pain, caregivers, qualitative analysis

## Abstract

**Background:**

Increasing the access to and improving the impact of pain treatments is of utmost importance, especially among youths with chronic pain. The engagement of patients as research partners (in contrast to research participants) provides valuable expertise to collaboratively improve treatment delivery.

**Objective:**

This study looked at a multidisciplinary exposure treatment for youths with chronic pain through the lens of patients and caregivers with the aim to explore and validate treatment change processes, prioritize and develop ideas for improvement, and identify particularly helpful treatment elements.

**Methods:**

Qualitative exit interviews were conducted with patients and caregivers at their discharge from 2 clinical trials (ClinicalTrials.gov NCT01974791 and NCT03699007). Six independent co-design meetings were held with patients and caregivers as research partners to establish a consensus within and between groups. The results were validated in a wrap-up meeting.

**Results:**

Patients and caregivers described that exposure treatment helped them better process pain-related emotions, feel empowered, and improve their relationship with each other. The research partners developed and agreed upon 12 ideas for improvement. Major recommendations include that pain exposure treatment should be disseminated more not only among patients and caregivers but also among primary care providers and the general public to facilitate an early referral for treatment. Exposure treatment should allow flexibility in terms of duration, frequency, and delivery mode. The research partners prioritized 13 helpful treatment elements. Most of the research partners agreed that future exposure treatments should continue to empower patients to choose meaningful exposure activities, break long-term goals into smaller steps, and discuss realistic expectations at discharge.

**Conclusions:**

The results of this study have the potential to contribute to the refinement of pain treatments more broadly. At their core, they suggest that pain treatments should be disseminated more, flexible, and transparent.

## Introduction

### Background

Chronic pain is among the largest contributors to disability in children [1], and suboptimal responses to current treatments remain a challenge for researchers and practitioners [2,3]. Youths with chronic pain experience major barriers to accessing adequate pain treatment (eg, those owing to shortage of providers and geographical distance) [4,5]. When offered a multidisciplinary pain treatment, a substantial number of patients decline to participate [6,7]. The reasons for this decline are largely unknown. Although there is evidence to support psychological interventions in pediatric chronic pain populations [3], there is room for improvement to enhance pain outcomes and emotional functioning.

Rooted in the fear-avoidance model [8], a graded exposure treatment (GET) was designed to more explicitly target maladaptive mechanisms of pain-related fear and avoidance to improve the return to function. GET has been shown to be an effective treatment for adults with chronic pain [9]. However, GET was associated with higher dropout rates than traditional cognitive behavioral therapy [9]. GET demonstrated preliminary efficacy in youths with chronic pain (GET Living) in an initial single-arm trial [7]. By 3- and 6-month follow-ups, >80% of participants showed improvements in the primary outcomes of fear and avoidance and secondary outcomes of pain catastrophizing, pain intensity, and pain acceptance. Analysis of data collected in a subsequent randomized controlled trial (RCT) to evaluate GET Living in a larger sample in comparison with a traditional multidisciplinary pain management approach is still ongoing [10]. Treatment delivery in the RCT shifted to a web-based format during the COVID-19 pandemic [11], with data collection concluding in January 2022 (Simons, LE, unpublished data, January 2022). Although this shift rapidly dispelled distance barriers, new issues related to adequate treatment delivery in patient homes emerged.

In the planning of the next GET Living iteration, we faced several questions that are also asked in the broader literature: How can pain exposure treatments be improved to produce long-lasting effects? How can we ensure that patients receive and participate in pain exposure treatments on a larger scale? Although the COVID-19 pandemic forced us to take unusual pathways, is the remote delivery format something we want to continue? Therefore, we decided to take an intermediate step to engage with people with lived experiences before deciding which action should be taken next.

Co-design is a “meaningful end-user engagement in research design that includes instances of engagement that occur at all stages of the research process and range in intensity from relatively passive to highly active and involved” [12]. Patients with lived experiences are engaged as consultants or partners in the research process (in contrast to traditional research participants) with the aim of collaboratively improving treatment efficacy, relevance, engagement, and delivery [13-15]. Participatory paradigms can be situated in implementation science, improvement science, and citizen science, although they often lack explanatory theories and models [15,16]. For example, outcome domains informed by expert guidelines do not necessarily represent meaningful domains for those receiving the intervention [17,18]. Similarly, clinicians and researchers risk limiting themselves in their understanding of treatment mechanisms depending on their preferred theoretical model [19]. It is possible that the mechanisms targeted and assessed during GET do not adequately capture all that might change for an individual during exposure treatment. Thereby, patients and caregivers with lived experiences can provide valuable feedback about how to improve treatment and what specific treatment elements were helpful in promoting change.

### Goal of This Study

In this study, we partnered with patients and caregivers who had previously received GET Living treatment [20]. From an improvement science perspective [15], our aims were to (1) explore and validate treatment change processes, (2) prioritize and develop ideas for improvement (ie, to refine the GET Living program for in-person and remote delivery), and (3) identify particularly helpful treatment elements to promote change.

## Methods

### Overview and Design

#### Overview

This project comprises two parts: (1) semistructured exit interviews and (2) co-design meetings. Qualitative exit interviews were conducted with patients and caregivers during a discharge session after they received the GET Living intervention as research participants. Subsequent co-design meetings were held with the patients and caregivers as research partners to refine the intervention in a formative research process.

#### Setting

This project involves 2 separate examinations of GET Living: one was a single-arm trial (Boston trial) and the other was an RCT (Stanford trial). The Boston trial (NCT01974791) used a sequential replicated and randomized single-case experimental design (SCED) with multiple measures evaluate the effect of GET on youths with chronic pain for the first time [7]. The Stanford trial (NCT03699007) used a 2-group RCT enhanced with SCED elements to compare GET Living with a traditional multidisciplinary pain management approach [10]. The former GET Living participants had a unique expertise in what it is like to undergo pain exposure treatment from a patient’s and caregiver’s perspective.

#### Recruitment

In the Boston trial, patients were recruited from the Pain Treatment Service at Boston Children’s Hospital between December 2013 and February 2017 (data collection was completed in January 2018). In the Stanford trial, patients were recruited from the Pediatric Pain Management Clinic at Stanford Children’s Health from January 2019 to May 2021 (data collection was completed in January 2022). Treatment providers referred patients to GET Living during clinic visits. A study flyer and additional brochures were also available in the patient waiting room for patients to self-refer to the study. Patients were deemed eligible to participate in GET Living if they were aged 8 to 17 years, had a diagnosis of chronic pain, had moderate to high pain-related fear, and had moderate to high functional disability [7,10].

### Part 1: Qualitative Exit Interviews

#### Goals and Overview

Interview data were analyzed to identify themes related to treatment change processes. In addition, interviews were conducted to create a pool of ideas for intervention improvement and helpful treatment elements, which were later ranked and discussed in the co-design meetings.

#### Interviewed Patients and Caregivers

Only the patients and caregivers who completed all treatment sessions were included in the qualitative analysis to ensure that the data were reflective of the entire treatment experience. The interview that was conducted with a patient and their caregiver who withdrew their participation was excluded. Both the patient and caregiver felt that the treatment’s focus on pain and anxiety was not a good fit. In the Boston trial, 26 interviews of patients and caregivers were analyzed. In the Stanford trial, 26 interviews of patients and caregivers who were randomized to the exposure intervention were analyzed. The patients and caregivers were interviewed separately. More details on the interviewed cohorts are presented in Table 1.

**Table 1:** Demographics and medical characteristics of youths who received GET Living^a^ in the first (n=26) and second (n=26) clinical trial.

Variable		Boston cohort	Stanford cohort
**Age (years)**			
	Values, mean (SD; range)	13 (3.12; 8-20)	14 (2.73; 8-18)
**Sex, n (%)**			
	Female	20 (77)	24 (92)
**Race, n (%)**			
	White	22 (85)	22 (85)
	Black or African American	1 (4)	2 (8)
	Multiracial	2 (8)	0 (0)
	Asian	0 (0)	1 (4)
	Unknown	1 (4)	1 (4)
**Parent marital status, n (%)**			
	Married	21 (81)	20 (77)
	Single	1 (4)	1 (4)
	Divorced or separated	4 (15)	4 (15)
	Widowed	0 (0)	1 (4)
**Pain diagnosis, n (%)**			
	Musculoskeletal	9 (35)	21 (81)
	Neuropathic	8 (31)	2 (8)
	Abdominal	6 (23)	3 (12)
	Headache	2 (8)	0 (0)
	Headache and musculoskeletal	1 (4)	0 (0)
**Duration of pain (months), n (%)**			
	Values, mean (SD; range)	22.6 (27.5; 1-65)	40.5 (37.1; 4-138)
**FDI^b^ at baseline, n (%) **			
	Values, mean (SD; range)	25.23 (10.3; 2-47)	23.15 (10.07; 4-42)
**Fear of pain (FOPQ^c^ total), n (%)**			
	Values, mean (SD; range)	50.96 (19.8; 9-82)	56.58 (15.9; 10-84)

^a^GET Living: graded exposure treatment for youths with chronic pain.

^b^FDI: Functional Disability Inventory.

^c^FOPQ: Fear of Pain Questionnaire.

#### Interview Guide

The semistructured exit interviews were conducted by research assistants during the discharge visit following the completion of GET Living. All research assistants were trained by the principal investigator LES. In the Boston trial, most interviews were conducted in person. In the Stanford trial, most interviews were conducted via phone or video calls. The patient and caregiver interview schedules both comprised 8 questions (Multimedia Appendix 1). The questions were intended to capture the positive (eg, question [Q] 1: “What did you like the best about GET Living treatment?” “What was the most helpful?”) and negative (eg, Q2: “What did not help?” “What would you change?”) experiences that the families had during their treatment. The participants were also encouraged to give their critical feedback through several questions (eg, Q3: “What do you wish you had known before starting GET Living treatment?”). Other questions targeted to capture treatment change processes, that is, the changes that the families experienced in themselves (eg, Q4: “What did you learn about yourself and your family in GET Living treatment?”). The interview schedule questions guided the conversation; however, consistent with the semistructured nature of the interview, the participants were also provided with space to share additional feedback about their experiences.

#### Analysis of the Exit Interview Data

Reflexive thematic analysis [21] was used to assess the participants’ perspectives and identify common themes across the interview data. Consistent with constructivist epistemology, reflexive analysis allows for the cocreation of knowledge between the participants and researchers. Subjectivity is not seen as a potential threat to the “*truthful*” or objective meaning of the data but is rather conceptualized as an analytical resource for data interpretation [22]. Data analysis was led by an investigator (LS) who was not involved in the data collection or intervention delivery. The analysis was conducted by following the 20-question guide by Braun and Clarke [22].

To begin data analysis, the investigator became familiar with the data by repeatedly and actively reading 12 fully transcribed interviews and listening to some randomly selected interviews. For the subsequent coding process, analysis was conducted on the audio recordings of interviews instead of the transcriptions to capture richer, more nuanced (eg, tone and affective aspects of responses) aspects of the participant responses. While listening, the investigator entered detailed notes of the codes for each interview into a comprehensive overview table. Relevant quotes were fully transcribed. Throughout the data analysis, the first author (LS) incorporated semantic features of the data (ie, explicitly stated ideas, concepts, meanings, and experiences) as well as latent features (ie, implicit meanings underlying explicit statements) when defining codes and themes. The generated codes were then clustered into candidate themes. This analytical process focused on the development of themes related to treatment change processes throughout the GET Living program. Theme identification occurred through an iterative process, whereby 2 authors (LS and LES) identified and refined codes and illustrative quotes until deep and nuanced themes regarding change processes were developed. Interview data regarding particularly helpful elements and ideas for improvement were organized into topic summaries (in comparison with fully developed themes). These topics summaries were used as a starting point to facilitate ranking and discussion in the subsequent co-design meetings. They will be presented when describing the results of the co-design meetings.

### Part 2: Co-design Meetings

#### Goals and Overview

The purpose of the co-design meetings was to validate the developed themes related to treatment change processes (eg, regarding their meaningfulness) and reach a consensus regarding important ideas for intervention improvement and key treatment elements. Consensus was established in 6 independent co-design meetings (ie, the nominal group technique) held as 3 parallel meetings with patients and caregivers. This allowed us to establish consensus within groups (ie, consensus in 1 group) and between groups (ie, consensus in multiple groups) as an estimate of the representativeness of the opinions expressed. Patients and caregivers served as ad hoc consultants [15] and were compensated for their efforts (US $30 per hour). Their role was to validate the research findings of the previous thematic analysis and to provide feedback about the GET Living treatment from the receiver’s end [15]. The procedures were preregistered in the Open Science Framework [23]. The GRIPP2 (Guidance for Reporting Involvement of Patients and the Public) checklist for patient and public participation in research guided quality reporting of the study results [24].

#### Patient and Caregiver Research Partners

Patients and caregivers who were randomized to the exposure treatment arm of the GET Living RCT (Stanford trial), including treatment completers and dropouts, were invited as research partners. Approximately one-third of the people invited accepted the invitation (10/33, 30% patients; 14/33, 42% caregivers). Research partners attended 1 of the 6 independent co-design meetings with parallel meetings for patients (meeting 1a: 4/10, 40%; meeting 2a: 3/10, 30%; meeting 3a: 3/10, 30%) and caregivers (meeting 1b: 4/14, 29%; meeting 2b: 5/14, 36%; meeting 3b: 5/14, 36%). All research partners were invited to a final wrap-up session (5/10, 50% patients and 8/14, 57% caregivers). More details on the research partners who attended the meetings are presented in Table 2.

**Table 2:** Demographics and pain characteristics of the patient (n=10) and caregiver (n=14) research partners who participated in the co-design meetings.

Variables			Youths with chronic pain		Caregivers
**Age (years)**					
	Values, mean (SD; range)	17 (2.4; 10-17)		49 (5.3; 35-55)	
**Sex, n (%)**					
	Female	10 (100)		12 (86)	
**Race, n (%)**					
	White	9 (90)		12 (86)	
	Black or African American	0 (0)		1 (7)	
	Asian	1 (10)		1 (7)	
**Ethnicity, n (%)**					
	Hispanic	0 (0)		2 (14)	
	Not Hispanic or Latino	10 (100)		12 (86)	
	Unknown	0 (0)		0 (0)	
**Pain diagnosis, n (%)**					
	Musculoskeletal	8 (80)		N/A^a^	
	Neuropathic	1 (10)		N/A	
	Abdominal	1 (10)		N/A	
**Duration of pain (months)**					
	Values, mean (SD; range)	38.27 (17.3; 14-66)		N/A	

^a^N/A: not applicable.

#### Procedure

The co-design meetings were scheduled for 2 subsequent calendar weeks (April 2022). The meetings were held via Zoom (Zoom Video Communications, Inc) and lasted approximately 120 minutes (including breaks). An optional web-based wrap-up meeting was held the following week (approximately 60 minutes). An overview of the procedure is provided in Multimedia Appendix 2. The meetings were moderated by CWH, LS, and LES.

Before the meeting, the research partners received a pre-engagement package with an outline of and the materials for the meeting. No preparation was required. At the beginning of the meeting, the research partners introduced themselves with ice-breaking tasks aimed at facilitating a good working atmosphere. Some ground rules were presented. Their role as research partners (as opposed to research participants) was highlighted.

The results of the thematic analysis of treatment change processes were then presented and discussed with the research partners to ensure that the identified themes were relevant and meaningful and to assess whether there were any important change processes missing from the established themes. The ideas for improvement collected during the semistructured exit interviews were then presented. The research partners were asked to rate the ideas using a Qualtrics (Qualtrics International Inc) survey. First, they were asked to select what they believed to be the 10 most important ideas out of the 48 ideas initially identified through the interviews. They were then asked to further refine their initial selection to identify the 3 most important ideas for improvement. The research partners were also encouraged to provide new ideas that were not found on the list as applicable. Once all the answers were collected, the results were shared with the group, and the research partners were asked to discuss the selections to ensure agreement among the retained items and address any differences of opinion regarding key recommendations. The same process was conducted to establish the most helpful out of 38 treatment elements that should be retained in future iterations of the intervention, and where applicable, the research partners were given intervention materials for review.

In the wrap-up meeting, action items from the co-design meetings were presented and finalized in a shared Word (Microsoft Corp) document. The research partners who could not attend the meeting were informed about the action items via email. They were asked to provide their written feedback within 2 weeks.

#### Evaluation of the Co-design Meetings

At the end of the meetings, all research partners completed module A of the Public and Patient Engagement Evaluation Tool [25]. Module A was developed to measure a 1-time engagement activity from a participant’s perspective. The module consists of 13 statements (eg, “I had a clear understanding of the purpose of the co-design meeting”), which the research partners were instructed to rate on a 5-point Likert scale (1=“strongly disagree,” 2=“disagree,” 3=“neither agree nor disagree,” 4=“agree,” and 5=“strongly agree”). The questionnaire also comprises 6 open-ended questions addressing key elements of quality public and participant engagement, including the integrity of design and process, influence and impact, participatory culture and collaboration, and common purpose. The questionnaire was used as a quality measurement of research partner engagement in the co-design meetings. In addition, we openly asked the research partners why they agreed to participate in the co-design meetings.

### Ethics Approval

Both trials received ethics approval from their respective institutional review boards (Boston: IRB-P0000727, and Stanford University: Protocol 39514). Before their participation, the patients and caregivers actively consented to take part in the respective clinical trial. The final version of the manuscript was sent to the patient and caregiver research partners. All research partners provided their consent for publication.

## Results

### Part 1: Results of the Thematic Analysis

**Figure 1:**
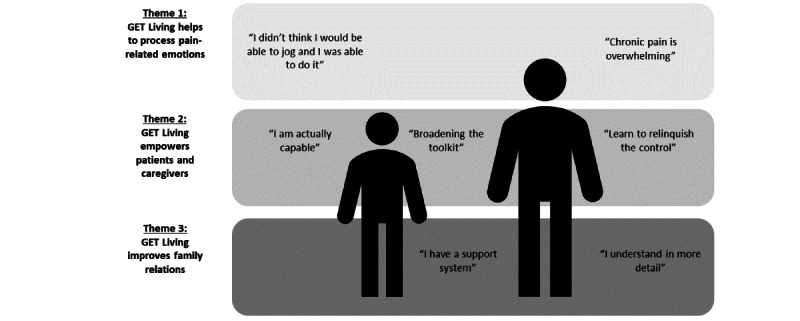
Subordinate themes describing treatment change processes experienced by the patient and caregiver. The developed themes are summarized on the left. The subthemes are displayed within the boxes, with the subthemes derived for the patients presented on the left (“I didn’t think I would be able to jog and I was able to do it” and “I am actually capable”), the subthemes derived for the caregivers presented on the right (“Chronic pain is overwhelming,” “Learn to relinquish the control,” and “I understand in more detail”), and the subthemes derived both patients and caregivers presented in the middle (“Broadening the toolkit” and “I have a support system”). GET: graded exposure treatment.

A total of 3 subordinate themes were generated from the reflexive thematic analysis of the exit interviews (Figure 1). These themes reflect the treatment change processes experienced by the patients and caregivers during the GET Living intervention. The themes were validated by the research partners in the co-design meetings.

### Theme 1: GET Living Helps Process Pain-Related Emotions

#### Overview

The first theme described how the patients and caregivers were better able to handle their pain-related emotions. Although the patients felt more confident in dealing with challenging situations, the caregivers had a space to process their own emotional struggles.

#### Patients: I Didn’t Think I Would Be Able to Jog and I Was Able to Do It

The patients learned through exposures that the experience was not as bad or challenging as they thought it would be. Overall, the patients described an emotional shift in their experience because it did not match their expected outcome:

It was probably the first time they told me to go for a jog, I didn’t think I would be able to do it, I got really scared but after I jogged with my mum it made me feel a lot better and I was able to do it and it made me happy.

B9, patient

Other patients and caregivers reported a change in their thinking:

Exposures were easier than I thought they would be.

B30, patient

The thought process going into it and getting those thoughts in check of how you can do things and not allowing misguided thinking to not allow you to do things that you can do.

S24, caregiver

There was a general shift in their perception of challenges. The patients also appeared to gain a sense of control:

I learned fear doesn’t control me, I can control it and I can control how to deal with it.

S15, patient

I learned to not be so afraid of things I loved to do. There are some challenges but I can get through them.

B20, patient

Those exposures really assisted her achieving goals she didn’t think she’d be able to make. And after that she was able to do more things.

S24, caregiver

Taken together, the patients realized that the situations they once feared were not as emotionally challenging or difficult as they expected them to be. On the basis of this experience, the patients seemed to see challenges as more approachable and manageable:

#### Caregivers: Chronic Pain Is Overwhelming

The caregivers became more aware of the overall experience of managing chronic pain and its impacts on the entire family:

Chronic pain is so overwhelming and such a challenge. Not only for the person in pain but the entire family really suffers from that...I don’t think I recognized how bad it was until I got into the program.

B19, caregiver

I learned that my pain impacted everyone in my family not just me, so like, the pain, I might feel it but everyone else can experience it too.

B29, patient

The caregivers had room to express and process their own emotional struggles:

And some were just the sad or grieving things. Especially her age going into young adulthood.

S46, caregiver

And [clinicians] said, “Your daughter’s gonna be okay. Her pain is real, but she’s gonna be ok.” That was really important for me to hear that. Finally, someone put it all together and made me feel like, okay she’s not gonna break.

GS33, caregiver

Some even expressed a newly found admiration toward their children:

So, I guess I admire kids and people who get through the pain somehow, and it’s without a break and they still manage. I guess my admiration for [child] and for people who’ve experienced that has increased.

B33, caregiver

And then, you know, I knew that [child] had it in him that he could push himself. I just think he needed help kinda pushing himself past that initial pain.

S9, caregiver

Taken together, the overwhelming experience of living with chronic pain was felt by the entire family. The caregivers mostly expressed feeling anxious or sad for their child; however, they were able to shift their perception by reinterpreting this struggle as a strength. Instead of feeling sad or anxious, they expressed an admiration for their children for handling painful situations.

### Theme 2: GET Living Empowers Patients and Caregivers

#### Overview

The second theme described how the patients and caregivers felt empowered during treatment. Whereas the patients experienced becoming more confident, the caregivers gave their children more space to handle difficult situations by themselves. Both felt that they learned concrete strategies for navigating difficult situations.

#### Patients: I Am Actually Capable

The patients’ experiences changed their perceptions of themselves. Many patients felt empowered and more confident. Some patients learned that they were capable of doing things despite their pain:

The only thing stopping me was myself...Well of course it was my back pain and all that. But I kind of held on to my back pain a little too much, for a little bit too long.

S33, patient

I learned that because I’ve been in a lot of pain and I put things off, that I am actually capable of doing a lot more.

B25, patient

I also learned that I can do anything even with my neck pain.

S24, patient

Other patients expressed a more generalized sense of being capable:

I am stronger than I thought I was.

B31, patient

I got more confident and stronger doing all the activities.

B22, patient

I learned that I was more determined and stronger than I thought.

S17, patient

Taken together, the patients changed their self-perception and appeared to be more confident in their ability to handle difficult situations in the face of pain and other challenges.

#### Caregivers: Learn to Relinquish the Control

The caregivers came to understand how to balance control and letting go. Some caregivers expressed that they could better see the benefits of giving their children more opportunities to handle their pain by themselves:

As parents, we do want to help out and control as much as we can, and to some extent I do still believe that we should be looking out for each other, you know, trying to prevent them from having pain, if it’s possible. But if it’s, in this kind of situation with the chronic pain thing, you learn to relinquish the control more and give them more options to handle it themselves.

S3, caregiver

I learned that [child] can be a lot more independent than I sometimes give him credit for. So sometimes I have to ease off in helping him. So, I think I learned that it is okay to let him tumble through something because then he will feel like he really did it himself.

S60, caregiver

Other caregivers reported that they became more aware of the negative effects of being overly controlling:

Well obviously, that we were holding her back from trying new things and not presenting things that would challenge or take her outside of her comfort zone. And we didn’t realize what we were doing.

B19, caregiver

I learned that I can be pretty intense and anxious which contributes to my child’s troubles or doesn’t help her cope. I learned to be more relaxed. Was too rigid before, let go of that now.

B5, caregiver

Generally, the caregivers were able to hold back on responding with their initial reaction to better respond to the specific needs of their children:

For me it was really about my own responses to her and how to control my responses and be more understanding of where she was at. I was so caught up in my own being tired and stuff that I was going through. I really had the opportunity to stop and take a look and understand how much she was impacted and how I could really help her.

B19, caregiver

Taken together, the caregivers were better able to control their initial reactions. After reflecting on the consequences of their own behavior, many caregivers said that they needed to relinquish some control to empower their children to manage challenging situations independently.

#### Patients and Caregivers: Broadening the Toolkit

The patients and caregivers broadened their knowledge of concrete strategies to better cope and live with chronic pain:

But also offering additional strategies for feeling like we don’t have to be helpless in the face of the pain when it’s severe. I think that was super helpful to both of us.

S22, caregiver

I liked the emphasis on creating a sense of confidence and broadening the toolkit for dealing with pain and the headaches. Kind of finding ways to carry forward. To make life worth living, kind of, you know, preserve some quality of life.

S22, caregiver

With strategies of knowing how to cope with pain, okay, I am gonna be able to approach those challenges of life more so because I have that tool under my belt that says, oh you are hurting today, you are not having a good day, how are you gonna get going and in the end come out the other side successful.

B23, caregiver

The patients learned to break down activities in pursuit of long-term goals:

I learned that taking a [small] step at a time can help me improve so much more than trying to take a big step.

B36, patient

I learned to just make accommodations instead of stopping the activity altogether.

B30, patient

I think her learning how to set goals that are achievable and measurable. And for her to be able to make them so that they are realistic. So, it was really her individualized goals. She made them up and she decided with the team where her values were.

B19, caregiver

The caregivers felt that they had solid action plans to encourage activities:

To give me a bit more vocabulary or instructions how to talk about things, like...“remember you wanted to do this because of the goals you set for yourself.”

S60, caregiver

And maybe just validate that we get it and when she does something that we recognize that. It does really help because when you are in the middle of it, you don’t really think about how we are going to react and it changes how she feels.

B19, caregiver

It empowered us as parents to say, we know your pain is real, we know it might cause a little bit of back pain, and you can take breaks. It gave us strategies for what we can say and what we can do to help encourage her still do her everyday activities.

S33, caregiver

Taken together, the patients and caregivers learned concrete strategies to navigate through difficult situations. These strategies helped reduce feelings of helplessness in both the patients and caregivers.

### Theme 3: GET Living Improves Family Relations

#### Overview

The third theme described how GET Living helped improve the relationship between patients and their caregivers. Being able to better understand the complexity of chronic pain, the caregivers were more able to validate their child’s experiences and felt closer to them. The patients also indicated that they felt more supported by their caregivers.

#### Caregivers: I Understand in More Detail

The caregivers better understood their child’s pain experience in their day-to-day difficulties:

I knew that she was hurting every day and that lots of things were difficult for her, but I think that I understand in more detail that even simple tasks, how and why they are difficult for her.

S57, caregiver

[Clinicians] taught me a lot regarding just [child]’s pain and how it can really, I don’t know, change her behavior. In that if [child] is grumpy or tired. I never associated the pain with her emotions before, neither did my husband. So, it was really eye opening for us to understand the correlation.

S24, caregiver

The caregivers also understood the driving mechanisms of pain chronicity in more detail:

I was also kind of surprised in the session when he was doing the soccer practice because he kind of attributed the time when all his leg pain started with soccer, even though soccer did not, you know, cause it.

S60, caregiver

It took away my anxiety that it will hurt, but it won’t harm her. The program made her try something. And some of the things she did, I knew that she would hurt herself. Not harm herself, but hurt herself.

B19, caregiver

The caregivers also reported being better able to validate the experiences of their child:

It never dawned on me before about how [child] could be feeling about this because no one can see it. And we just gave her a hard time about school and that she is not feeling it. And sometimes with that they have to keep validating it and hold on to it. And maybe just validate that we get it and when she does something that we recognize that. It does really help because when you are in the middle of it, you don’t really think about how we are going to react and it changes how she feels.

B19, caregiver

My mom and dad...actually knew how I feel now and what I was going through.

B20, patient

Taken together, the caregivers became more aware of the difficulties of their children. They better understood the impact of chronic pain on pain-related disability and distress. The caregivers also became aware of the driving mechanisms of chronic pain, including emotional responses and misattributions. This allowed them to validate their child’s experience more.

#### Patients and Caregivers: I Have a Support System

The patients and caregivers reported that GET Living fostered improved family connections. The patients became more aware that they were not alone because they had their caregivers and families to support them:

And that my family can help me do whatever, that I don’t just have to rely on myself to help these things. I have a support system.

S33, patient

I learned that my family are very enthusiastic and willing to do those things with me.

S22, patient

And my family, I think, learned if I am in pain how they can help me deal with it.

S15, patient

The caregivers also felt a closer connection with their children:

We kind of had a better connection than we did before. Not that we had a bad connection, it’s just the drives to the sessions.

S9, caregiver

I felt like some of the sessions led to more discussions with [child] and I afterwards, like I felt that there were certain things, like as a mother daughter, that it was positive.

S46, caregiver

Taken together, the relationships between the patients and their families improved. While the patients felt supported, the parents felt a closer connection with their children.

### Part 2: Results of the Co-design Meetings

#### Ideas for Improvement

A total of 12 ideas for improvement were prioritized in multiple groups (ie, between-group consensus) and are presented in Table 3. The ideas were organized based on the degree of consensus between the groups. Five ideas that were prioritized by within-group consensus are presented in Multimedia Appendix 3.

Interestingly, these improvement ideas were not specific to exposure treatments and could be applied to any form of behavioral or physical pain treatment. For example, the research partners agreed that pain exposure treatment should be disseminated more. There was absolute consensus (consensus in 6/6, 100% co-design meetings) that pain exposure treatment should use patient testimonials to (1) provide patients with narratives of how other patients are dealing with similar difficulties, (2) inform future patients about what treatment will be like, (3) provide a role model, and (4) promote positive expectations. Most research partners also agreed that more efforts should be made to create awareness among the general public and primary care providers to facilitate an early referral for treatment (consensus in 4/6, 67% co-design meetings).

**Table 3:** Ideas of improvement developed and agreed upon in the co-design meetings using the nominal group technique (consensus in multiple groups)^a^.

An ideal GET Living^b^ program would...	Concrete ideas	Consensus between groups (n=6), n (%)
...inform what the treatment will be like and promote positive expectations	Patient and caregiver testimonials (eg, videos) to see other patients dealing with similar difficulties, provide a role model, better understand what the treatment will be like, transmit hope for future patients Clarify that the treatment aims to increase activity and explain the role of PT^c^ (compared with traditional PT)	6 (100)
...start earlier with more interdisciplinary exchange	More awareness of the program through posters, flyers, websites, and social media Campaign educating primary care providers about this modality as a treatment option to facilitate early referral More exchange and referral between providers (eg, to discuss treatment progress)	4 (67)
...allow for more flexibility	Adapt the duration, frequency, and content to the momentary pain level or energy of patients Flexible web-based sessions when pain level is too high	4 (67)
...be also offered remotely with optional in-person meetings	Optional in-person meetings to build trust and help patients get a better diagnostic view of the exposure activities Help overcome technical barriers (eg, send treatment materials at home and provide Wi-Fi booster)	3 (50)
...add booster sessions	Combination of structured and client-lead booster sessions (eg, reminder of the core treatment elements and think together how they can be applied to real life)	3 (50)
...be honest that becoming better is not easy but it is a process	Emphasize that treatment provides long-term strategies Provide feedback on progress (especially little steps) as a motivator Help to find the balance of being challenged but not overwhelmed	3 (50)
...have the patient decide if parent should participate in treatment	Discuss with patients whether caregivers should join the treatment Optional patient-only sessions	3 (50)
...be offered also to patients over 18	Support in an especially vulnerable phase of transition into young adulthood (eg, decision on the future) on top of chronic pain	2 (33)
...enable patients to meet other patients	Platform to exchange information with other patients of similar age (eg, ages of 8 to 12 years and ages 13 to 17 years) Open coffee hours via Zoom (eg, once per month) Web-based education sessions or booster sessions together with other patients	2 (33)
...enable parents to meet other parents	Platform for support and exchange (eg, see other families who go through the same thing and think together how to positively influence family dynamics)	2 (33)
...include more complex pain ratings	Description of end points (eg, developing individualized reference points at the beginning of treatment) Body map to describe pain localization and give differential pain ratings for different locations	2 (33)
...be adapted to other symptoms experienced besides pain	For example, adapt exposure activities to additional symptoms of dizziness Editable worksheets to personalize exposure activities	2 (33)

^a^Ideas for improvement are organized according to the degree of consensus between groups.

^b^GET Living: graded exposure treatment for youth with chronic pain.

^c^PT: physical therapy.

#### Helpful Treatment Elements to Promote Change

A total of 13 treatment elements were considered helpful in promoting change in multiple groups (between-group consensus; Table 4). For a clear overview, helpful treatment elements are organized by treatment phase. Seven treatment elements that were considered helpful by only the members of 1 group are presented in Multimedia Appendix 4 (within-group consensus).

In general, the research partners appreciated the understanding attitude of clinicians, personalization of treatment through the pursuit of individualized goals, education about chronic pain, encouragement of activities, and discussion of realistic expectations at discharge. For example, during the phase of goal setting, a majority of the research partners agreed that future exposure treatments should continue to empower patients to be “in charge” to choose meaningful exposure activities (consensus in 5/6, 83% co-design meetings), break long-term goals into smaller steps (consensus in 5 of the 6 co-design meetings, 83%), and help patients become aware of their own values and motivators (consensus in 2/6, 33% co-design meetings).

**Table 4:** Most helpful treatment elements agreed upon in co-design meetings using the nominal group technique (consensus in multiple groups)^a^.

Treatment phase and future GET Living^b^ programs (regardless of the delivery format) should continue to...		Consensus between groups (n=6), n (%)
**Building rapport**		
	...combine pain psychology and physical therapy	3 (50)
	...transmit the feeling that it is possible to deal with pain	2 (33)
	...offer validation and understanding of patients’ situation	2 (33)
**Goal setting**		
	...empower patients to be “in charge” to choose meaningful activities	5 (83)
	...distinguish between short-term and long-term goals	5 (83)
	...help patients become aware of their values and motivators	2 (33)
**Education**		
	...reflect on triggers of pain and anxiety	3 (50)
	...distinguish between short-term and long-term solutions	2 (33)
	...include the exposure graphs	2 (33)
**Exposures**		
	...encourage activities allowing for breaks and a slow pace	3 (50)
	...teach the use of facilitators	3 (50)
	...include the WILD^c^ scale	2 (33)
**Discharge**		
	...discuss realistic expectations at discharge (eg, discuss coping with pain flare-ups)	6 (100)

^a^Treatment elements that were considered helpful are organized by treatment phases.

^b^GET Living: graded exposure treatment for youth with chronic pain.

^c^The WILD scale assesses a patients’ perceived Willingness, Importance, Likelihood of Success, and Difficulty with regard to the chosen exposure. The scale is completed before and after exposure [10]. The WILD scale of an example patient can be found in Multimedia Appendix 5.

#### Evaluation of the Co-design Meetings

Overall, the co-design meetings were evaluated as good, with mean values being consistently at the upper end of the agreement scale (Tables 5 and 6). The research partners felt that the co-design meeting was a good use of their time, that they were able to contribute, and that they were confident that the meeting’s goals were achieved.

**Table 5:** Quantitative results of the Public and Patient Engagement Evaluation Tool^a^.

Item			Patients, mean (SD; range)		Caregivers, mean (SD; range)
**Communications and supports for participation**					
	I had a clear understanding of the purpose of the co-design meeting.	4.3 (0.95; 2-5)		4.4 (0.63; 3-5)	
	The supports I needed to participate were available (eg, travel, childcare, etc).	4.5 (0.71; 3-5)		4.4 (0.76; 3-5)	
	I had enough information to contribute to the topic being discussed.	4.6 (0.52; 4-5)		4.6 (0.51; 4-5)	
**Views and perspectives**					
	I was able to express my views freely.	5 (0; 0-5)		4.9 (0.36; 4-5)	
	I feel that my views were heard.	4.9 (0.32; 4-5)		4.9 (0.36; 4-5)	
	A wide range of views on the topics discussed was shared.	4.5 (0.53; 4-5)		4.7 (0.47; 4-5)	
	The individuals participating in this co-design meeting represented a broad range of perspectives on the topic.	4.4 (0.7; 3-5)		4.5 (0.52; 4-5)	
**Impacts and influence of engagement initiative**					
	I think that the co-design meeting achieved its objectives.	4.7 (0.48; 4-5)		4.5 (0.52; 4-5)	
	I am confident the input provided through this initiative will be used by Biobehavioral Pediatric Pain Lab.	4.5 (0.71; 3-5)		4.6 (0.65; 3-4)	
	I think the input provided through this activity will make a difference to the work of the Biobehavioral Pediatric Pain Lab.	4.6 (0.7; 3-5)		4.6 (0.5; 4-5)	
**Final thoughts**					
	As a result of my participation in the co-design meeting, I am better informed about the Biobehavioral Pediatric Pain Lab.	4.4 (0.7; 3-5)		4.1 (0.77; 3-5)	
	Overall, I was satisfied with this engagement initiative.	4.6 (0.52; 4-5)		4.7 (0.47; 4-5)	
	This engagement initiative was a good use of my time.	4.7 (0.48; 4-5)		4.6 (0.65; 3-5)	

^a^1=strongly disagree, 2=disagree, 3=neither disagree or agree, 4=agree, and 5=strongly agree.

**Table 6:** Qualitative results of the Public and Patient Engagement Evaluation Tool plus reasons for participation.

Open-ended questions	Patients	Caregivers
What else would you like us to know about how your participation in the co-design meeting was supported?	Felt supported	Easy web-based format with handouts given before Accommodating and flexible scheduling
What else would you like us to know about how you were able to share your views?	Everyone was easy to talk to Everyone brought different perspectives and life experiences, which shaped their advice and made the discussion interesting	Easier to share openly via Zoom
What else would you like us to know about the influence you think the co-design meeting will have?	N/A^a^	Input may guide future improvements of an already great program
What were the strengths of the co-design meeting?	Everyone was nice and supportive Ability to contribute perspective on what to improve upon Engaging and friendly leaders Materials provided in advance Surveys helped facilitate discussion	Leaders were open and understanding, and our opinions were validated Valuable to hear other perspectives from other patients Able to voice concerns and connect with and hear the opinions of other caregivers Smaller groups allowed for everyone’s voice to be heard Breakout rooms so that youths and caregivers could discuss separately Informal nature allowed for comfortability
What could be improved about the co-design meeting?	More icebreakers and introductions to meet the others in the meeting Allow the patients to talk freely about their experience without structure to allow for suggestions that the researchers had not proposed and to allow the patients to connect with one another	Would have liked a time to share freely without any structure
What else would you like us to know about your experience with the co-design meeting?	It was a great way to allow the past patients to feel more included and important	Allowed caregivers to hear others’ experiences and thoughts
Why did you agree to be part of the co-design meetings?	Wanted voice to be heard Wanted to give back to a program that helped me Wanted to help improve the program for others with chronic pain	To help others with chronic pain To give back and help this program This study was very important to our family Wanted to share my ideas for improvements

^a^No one answered the question.

## Discussion

### Principal Findings

#### Overview

This study looked at a multidisciplinary exposure treatment for youths with chronic pain through the lens of patients and caregivers. First, qualitative analysis of exit interviews conducted with patients and caregivers after they received the GET Living intervention explored the treatment change processes. Second, co-design meetings with patients and caregivers as research partners aimed to refine the GET Living intervention. The implications of both aspects are discussed in the subsequent sections.

#### Treatment Change Processes: What Changes and How?

The qualitative analysis revealed a wide range of treatment change processes, indicating that what happens within patients during treatment is complex and difficult to describe from a single theoretical lens [19]. The patients and caregivers described that the exposure treatment helped them to (1) better process pain-related emotions, (2) feel empowered, and (3) improve their relationship with each other. The elements of these reported changes align with different theoretical models. In line with the inhibitory learning approach [26,27], the patients experienced a violation of their expectations, wherein feared situations were not as emotionally challenging or difficult as expected they them to be. By contrast, the caregivers reported a reduction in protective behavioral responses when they felt more in control of their emotional distress, which, in turn, empowered the patients to handle difficult situations themselves. This aligns with the theoretical assertions of the interpersonal fear–avoidance model [28,29]. In addition, the patients reported changes that are considered resources according to the resilience-risk model [30]. On an individual level, the patients reported improved self-esteem (“I am actually capable”). In terms of their family and social environment, they felt more supported (“I have a support system”). Consistent with the interpersonal process model of intimacy, the patients and caregivers experienced an increase in intimacy and improvement in their relationship when the caregivers were better able to understand and validate the patients’ pain experience [31]. Looking through the lens of self-determination theory [32], the treatment might have satisfied the need for autonomy and competence (eg, by having patients be “in charge,” which was ranked as a particularly helpful treatment element), which facilitated goal pursuit despite chronic pain and an increased a sense of confidence. The patients also felt more supported, indicating satisfaction in the need for relatedness. However, the patients wished to extend this support to their peers with chronic pain. Altogether, the present results underscore the need for a more holistic approach to understand the full complexity of treatment change processes within patients and in their interaction with their social environment. Future research using and combining contemporary quantitative methods (eg, ambulatory assessments, network analyses, and SCEDs) could use the present findings to flexibly and rigorously study treatment change processes from idiographic and nomothetic perspectives [33].

#### Refinement of Pediatric Pain Treatments: What Should We Do Better?

The research partners prioritized 13 core treatment elements that were helpful in promoting change. This feedback can directly inform clinicians which specific behaviors and techniques are perceived as impactful. This is informative for clinicians in general but especially in settings with time constraints. For example, a majority of the research partners agreed that future exposure treatments should continue to assist patients in finding and pursuing meaningful goals. This recommendation agreed with pain scientists, who advise combining exposure treatment with clarification interventions to identify personal goals and goal conflicts [34]. Such techniques could also have the potential to ameliorate other behavioral programs. However, future research should systematically investigate the benefits of these techniques (eg, improving outcomes or facilitating the transfer of skills to daily life).

The research partners also agreed upon 12 ideas for improvement. At their core, these ideas suggest that pain treatments should be disseminated more, flexible, and transparent. The research partners advised that there should be a platform for exchange between people with lived experiences and that the complexity of the individual pain experience should be acknowledged. To our surprise, most ideas were not specific to the content and refinement of exposure treatment; instead, they could inform the implementation of behavioral or physical pain treatments more broadly.

The research partners conveyed that increasing the access to and dissemination of pain psychology treatment is of upmost importance, a message also building momentum among pain scientists [35]. At the receiver’s end, the research partners recommended to better clarify the role of psychological interventions in the context of a multidisciplinary pain treatment approach. Thereby, they came up with creative ideas such as video testimonials or advertising campaigns to clarify treatment aims and promote positive expectations. At the same time, the research partners also suggested better acquainting other treatment providers with this treatment option to facilitate early referral. In addition, they considered an increase in flexibility (eg, in terms of session duration, frequency, content, and delivery format depending on momentary pain level) a promising step toward improvement. Although shifting plans based on pain levels stands in contrast to pain scientists advocating that time and quota–contingent treatment plans are preferred over pain-contingent plans [36], it introduces an important consideration for pragmatic implementation in real life. Momentarily scaling back an activity versus rigid adherence to a plan could ultimately provide the flexibility needed to reach the long-term goal of greater life engagement and functionality.

The research partners considered the remote delivery format with optional in-person check-ins (eg, to build trust) as promising beyond the pandemic, which aligns with initiatives underway in the pain treatment field, as the pandemic has accelerated the dissemination of remotely delivered pain management services [37]. Continuing this path might contribute to a greater dissemination of pain psychology treatments, especially among youths. Remotely delivered treatments might also be beneficial for other behavioral or physical treatments (eg, to facilitate integration into daily life). Moreover, the research partners wanted more support in transferring and maintaining learned strategies (eg, via booster sessions). This request suggests potential ways to address the issue that the effects of pain psychology treatments are often not stable over time [3]. Altogether, the research partners created an abundant set of ideas focused on improving the delivery of pain treatments. From a human-centered design perspective, the present results specify the needs of patients and caregivers [38]. Future research could use these ideas to investigate whether tailoring implementation strategies to end users’ needs relates to better behavioral (eg, penetration) and perceptual (eg, acceptability) implementation outcomes [39]. For example, it would be interesting to see whether tailoring implementation strategies for pain treatments results in fewer people declining to participate and fewer dropouts.

### Strength and Limitations

We provided an in-depth analysis of a specialized multidisciplinary exposure treatment for youths with chronic pain. Although we provided an overview of the change processes experienced by patients and caregivers, we could establish whether they contributed to the overall improvement (eg, increase in physical activity and school performance) using the methods we adopted. The patients and caregivers did not report having experienced treatment side effects, although it should be noted that we included only treatment completers in the thematic analysis. We also did not explicitly ask about treatment side effects. Our findings may not be generalizable to other behavioral pain treatments or pain populations, although it is likely that the fundamental processes identified are cross-cutting. Multiple co-design meetings allowed us to establish consensus within and between groups. This can be taken as an estimate of the representativeness of the expressed opinions. However, the included research partners were not representative, even of the US population, in terms of underrepresented groups, with most research partners being White and female. The study was conducted within the US health care system, and the results may not be generalizable to other health care systems and countries. In Germany, for example, the distances between patients’ homes and outpatient pediatric care centers are smaller, and the acceptance of internet-delivered treatments is rather low [40]. We did not present differential consensus ratings for patient and caregiver research partners because they were largely congruent. Only the involvement of caregivers during treatment was a critical point, where although the patients wanted less involvement, their parents wanted more involvement. The compromise developed in the wrap-up meeting was to negotiate the amount of involvement at the beginning of treatment (also depending on the patient’s age) and offer patient-only sessions.

### The Future of GET Living

The GET Living team is poised to iterate and implement the advice learned from the patients and caregivers as research partners. Planned modifications span 3 key domains: publicity and education, treatment delivery, and supporting families after treatment completion. We intend to develop video testimonials that weave in the ingredients the patients and caregivers defined as essential, namely the opportunity to process pain-related emotions, feeling empowered, and improving their relationship with one another. In addition to patient and parent testimonials, we would like to roll out an advertising campaign that targets both patient families and providers regarding the role of psychology in pain treatment and in some instances, more specifically, the GET Living treatment approach. These 2 publicity and education initiatives will better elucidate treatment aims, address misconceptions, and cultivate positive expectations regarding treatment. In the realm of treatment delivery, we have demonstrated in our latest clinical trial the capability to deliver GET Living remotely [11], and a clinical trial to implement a digital exposure intervention is underway ([41]; NCT05079984). Finally, we aim to devise approaches that will lead to lasting positive effects. We envision integrating booster sessions up to 1 year after treatment completion, potentially a combination of in-person and remotely delivered sessions. Moreover, we can leverage our developing digital content to push resources to patient families over time and provide a library of tools accessible long after treatment completion. Altogether, these research partner–guided changes will undoubtedly improve engagement and outcomes among youths with chronic pain. For the future of GET Living, we plan to establish patients and caregivers as standing members of an advisory board to facilitate a closer collaboration with them during the next iteration of GET Living.

### Conclusions

This study has revealed several powerful implications that should be considered in future treatments and studies. The exit interviews with the patients and caregivers demonstrated the full complexity of treatment change processes. The research partners agreed that pain exposure treatment should be disseminated more, flexible, and transparent. These implications would not have been revealed if only traditional outcome and facility measures had been used. The clear and meaningful outcomes of this study strongly support the involvement of patients and caregivers in pain treatment manual developments and pain study designs.

## Appendix

Multimedia Appendix 1 List of semistructured interview questions.

Multimedia Appendix 2 Co-design meeting schedule.

Multimedia Appendix 3 Ideas of improvement developed and agreed upon in co-design meetings using the nominal group technique (consensus in one group).

Multimedia Appendix 4 Most helpful treatment elements agreed upon in co-design meetings using the nominal group technique (consensus in one group).

Multimedia Appendix 5 The Willingness, Importance, Likelihood of Success, and Difficulty scale.

## Abbreviations

GET: graded exposure treatment
GRIPP2: Guidance for Reporting Involvement of Patients and the Public
RCT: randomized controlled trial
SCED: single-case experimental design
Q: question
