# Supplementary material for: Enhancing Exposure Treatment for Youths With Chronic Pain: Co-design and Qualitative Approach
Source: J Particip Med. 2023 Mar 9;15:e41292. doi: 10.2196/41292 (PMC10037174; doi:10.2196/41292)
Supplement: Multimedia Appendix 1 [file jopm_v15i1e41292_app1.pdf]

List of semi-structured interview questions

Child interview

Q1: What did you like best about GET Living treatment? What was the most helpful to you?

Q2: What did not help? What would you change?

Q3: What do you wish you had known before starting GET Living treatment?

Q4: What did you learn about yourself and your family in GET Living treatment?

Q5: What will help you to stay motivated to engage in challenging valued activities after GET Living treatment?

Q6: What obstacles do you anticipate, and how do you plan to handle these?

Q7: Would you recommend GET Living treatment to other kids with pain? Why or why not?

Q8: What advice would you give to a new patient just starting the GET Living treatment program?

Caregiver interview

Q1: What did you like best about GET Living treatment? What was the most helpful to you? What was most helpful to your child?

Q2: What did not help? What would you change?

Q3: What do you wish you had known before starting GET Living treatment?

Q4: What did you learn about yourself and your family in GET Living treatment?

Q5: 5. What do you think will help your child stay motivated to engage in challenging valued activities after GET Living treatment?

Q6: 6. What obstacles do you anticipate that you or your child may face after GET Living treatment?

Q7: 7. How do you view your role in helping your child to engage in challenging valued activities after GET Living treatment?

Q8: 8. Would you recommend the program to other families of children with pain? Why or why not?

Schemer L, Hess CW, Van Orden AR, Birnie KA, Harrison LE, Glombiewski JA, Simons LE. Enhancing exposure treatment for youth with chronic pain: A co-design and qualitative approach. J Particip Med. [doi: 10.2196/41292]
