# Supplementary material for: Enhancing Exposure Treatment for Youths With Chronic Pain: Co-design and Qualitative Approach
Source: J Particip Med. 2023 Mar 9;15:e41292. doi: 10.2196/41292 (PMC10037174; doi:10.2196/41292)
Supplement: Multimedia Appendix 2 [file jopm_v15i1e41292_app2.pdf]

Co-Design Meeting Schedule

Topic covered	Time	Specific tasks	Guiding questions
Treatment change processes within the GET Living program	15 min	Reviewing developed themes around treatment change processes	Are the developed themes meaningful?
Refinement of the GET Living manual	45 min	Reaching consensus and providing recommendations for improvement (for in-person and remote delivery)	Which ideas are particularly promising for the refinement of the GET Living program?
Identification of helpful treatment elements of the GET Living manual	30 min	Reaching consensus on particularly helpful treatment elements	Which treatment elements are most helpful to promote change?

Schemer L, Hess CW, Van Orden AR, Birnie KA, Harrison LE, Glombiewski JA, Simons LE. Enhancing exposure treatment for youth with chronic pain: A co-design and qualitative approach. J Particip Med. [doi: 10.2196/41292]
