# Supplementary material for: Enhancing Exposure Treatment for Youths With Chronic Pain: Co-design and Qualitative Approach
Source: J Particip Med. 2023 Mar 9;15:e41292. doi: 10.2196/41292 (PMC10037174; doi:10.2196/41292)
Supplement: Multimedia Appendix 3 [file jopm_v15i1e41292_app3.pdf]

Ideas of improvement developed and agreed upon in co-design meetings using the nominal group technique (consensus in one group)

**An ideal GET Living program would…**	**Concrete Ideas**
…provide education sessions in a smaller dose	• Provide educational information in a digital way leaving sessions for more active work;<br>• Formatting sessions to start with activity or trade back and forth with activities and talk
…allow animals (e.g., dogs) to help during exposure activities	• Engage patients with foster animals, adopt an animal to increase motivation
... ask more relevant daily questions	• Inclusion of open questions, more individualized questions
…practice the same exposure activity more often	• Implement more reminders (e.g., discuss the last exposure activities (last session or homework) at the beginning of each session)
…have more elaborate exposure activities, also outside the treatment facility	• Exposure activities in real life with Zoom support via smartphone

Schemer L, Hess CW, Van Orden AR, Birnie KA, Harrison LE, Glombiewski JA, Simons LE. Enhancing exposure treatment for youth with chronic pain: A co-design and qualitative approach. J Particip Med. [doi: 10.2196/41292]
