# Supplementary material for: Enhancing Exposure Treatment for Youths With Chronic Pain: Co-design and Qualitative Approach
Source: J Particip Med. 2023 Mar 9;15:e41292. doi: 10.2196/41292 (PMC10037174; doi:10.2196/41292)
Supplement: Multimedia Appendix 4 [file jopm_v15i1e41292_app4.pdf]

Most helpful treatment elements agreed upon in co-design meetings using the nominal group technique (consensus in one group)

Treatment phase	Future GET Living programs (regardless of the delivery format) should continue to…
Building rapport	…keep messaging of treatment aims consistent
	…give feedback about the progress (e.g., visualization through ratings)
Exposures	...include the activity ladder
	…have exposure activities tailored to level of functioning
	…include tracking watching to encourage to be more active
Inclusion of caregivers	…include caregivers in the treatment
	...reflect on the consequences a caregivers' behavior has on their child

Schemer L, Hess CW, Van Orden AR, Birnie KA, Harrison LE, Glombiewski JA, Simons LE. Enhancing exposure treatment for youth with chronic pain: A co-design and qualitative approach. J Particip Med. [doi: 10.2196/41292]
