# Supplementary material for: Enhancing Exposure Treatment for Youths With Chronic Pain: Co-design and Qualitative Approach
Source: J Particip Med. 2023 Mar 9;15:e41292. doi: 10.2196/41292 (PMC10037174; doi:10.2196/41292)
Supplement: Multimedia Appendix 5 [file jopm_v15i1e41292_app5.pdf]

WILD Scale

Activity:
Taking care of horses (e.g. cleaning hooves)

How will I practice it?
I will bend forward 4 times

How willing are you to have pain/distress and still do this activity?
If I want to get back on a horse, I must be able to take care of my horse. This is important to me!!

WILD Scale Ratings before after

W *Willingness- How willing are you to have pain/distress and still do this activity?*

Not at all willing (no way) _ _ _ _ _ _ _ _ _ _ _ _ _ _ _|_ _ x _ _ x _ _ _ _ _ _ _ _ _ Very willing

I *Importance- How important is this activity to you in your life?*

Not at all important _ _ _ _ _ _ _ _ _ _ _ _ _ _ _|_ _ _ _ _ _ _ _ _ x x _ _ _ _ Very Important

L *Likelihood of success- How likely is it that you will be successful with this activity?*

Not at all likely to succeed _ _ _ _ _ _ _ _ _ _ _ _ _ _ x|_ _ _ _ _ _ _ _ _ x _ _ _ _ _ Very likely to succeed

D *Difficulty- How difficult do you think this task will be?*

Very difficult, almost impossible _ _ _ _ _ _ _ _ _ _ _ _ _ _ _|_ x _ _ _ _ _ x _ _ _ _ _ _ _ Not at all difficult

***Exposure Action Plan (EAP)*: Circle at least two strategies to help you keep going, even if you get stuck**

- **Facilitators:** listening to my favorite song while cleaning the hooves and bending over
- **Stretches:** shoulders stretch
- **Movement break**
- **Take deep breaths**
- **Just get into it!**
- **Think helpful thoughts (hint – ask yourself the questions below!):**

My Mom would say: "You can do it"

If Lisa didn't have the confidence, I'd tell her she could do it.

When I bent over in the therapy session last week, nothing bad happened.

Why is it important? I want to go horse riding (!!)

? What would someone who cares about me say to help me to keep going?

? What would I say to a friend who was trying to do this activity?

? What happens most times that I do this activity?

? Why is this activity important to me?

Schemer L, Hess CW, Van Orden AR, Birnie KA, Harrison LE, Glombiewski JA, Simons LE. Enhancing exposure treatment for youth with chronic pain: A co-design and qualitative approach. J Particip Med. [doi: 10.2196/41292]
